# Cigarette smoke enhances oncogene addiction to c‐MET and desensitizes EGFR‐expressing non‐small cell lung cancer to EGFR TKIs

**DOI:** 10.1002/1878-0261.12193

**Published:** 2018-04-14

**Authors:** Chih‐Yen Tu, Fang‐Ju Cheng, Chuan‐Mu Chen, Shu‐Ling Wang, Yu‐Chun Hsiao, Chia‐Hung Chen, Te‐Chun Hsia, Yu‐Hao He, Bo‐Wei Wang, I‐Shan Hsieh, Yi‐Lun Yeh, Chih‐Hsin Tang, Yun‐Ju Chen, Wei‐Chien Huang

**Affiliations:** ^1^ Department of Life Science the iEGG and Animal Biotechnology Center National Chung Hsing University Taichung Taiwan; ^2^ Division of Pulmonary and Critical Care Medicine Department of Internal Medicine China Medical University Hospital Taichung Taiwan; ^3^ School of Medicine China Medical University Taichung Taiwan; ^4^ Graduate Institute of Basic Medical Science China Medical University Taichung Taiwan; ^5^ Graduate Institute of Cancer Biology China Medical University Taichung Taiwan; ^6^ The Ph.D. Program for Cancer Biology and Drug Discovery China Medical University and Academia Sinica Taichung Taiwan; ^7^ Department of Respiratory Therapy China Medical University Taichung Taiwan; ^8^ Hyperbaric Oxygen Therapy Center Department of Internal Medicine China Medical University Hospital Taichung Taiwan; ^9^ Graduate Institute of Biomedical Science China Medical University Taichung Taiwan; ^10^ Department of Medical Research E‐DA Hospital Kaohsiung Taiwan; ^11^ Department of Biological Science & Technology I‐Shou University Kaohsiung Taiwan; ^12^ School of Medicine I‐Shou University Kaohsiung Taiwan; ^13^ Center for Molecular Medicine China Medical University and Hospital Taichung Taiwan; ^14^ Department of Biotechnology College of Health Science Asia University Taichung Taiwan; ^15^ Research Center for New Drug Development China Medical University Taichung Taiwan

**Keywords:** benzo[α]pyrene, cigarette smoke, c‐MET, EGFR‐TKI, lung cancer

## Abstract

Cigarette smoking is one of the leading risks for lung cancer and is associated with the insensitivity of non‐small cell lung cancer (NSCLC) to epidermal growth factor receptor (EGFR) tyrosine kinase inhibitors (TKIs). However, it remains undetermined whether and how cigarette smoke affects the therapeutic efficacy of EGFR TKIs. In this study, our data showed that chronic exposure to cigarette smoke extract (CSE) or tobacco smoke‐derived carcinogen benzo[α]pyrene, B[α]P, but not nicotine‐derived nitrosamine ketone (NNK), reduced the sensitivity of wild‐type EGFR‐expressing NSCLC cells to EGFR TKIs. Treatment with TKIs almost abolished EGFR tyrosine kinase activity but did not show an inhibitory effect on downstream Akt and ERK pathways in B[α]P‐treated NSCLC cells. CSE and B[α]P transcriptionally upregulate c‐MET and activate its downstream Akt pathway, which is not inhibited by EGFR TKIs. Silencing of c‐MET reduces B[α]P‐induced Akt activation. The CSE‐treated NSCLC cells are sensitive to the c‐MET inhibitor crizotinib. These findings suggest that cigarette smoke augments oncogene addiction to c‐MET in NSCLC cells and that MET inhibitors may show clinical benefits for lung cancer patients with a smoking history.

**Abbreviations**
5‐mC: anti‐5‐methylcytosine
B[α]P: benzo[α]pyrene
CM: conditioned medium
CSE: cigarette smoke extract
DCR: disease control rate
DEPC: diethyl pyrocarbonate
DEPC: diethyl pyrocarbonate
HGF: hepatocyte growth factor
NC: nitrocellulose
NF‐κB: nuclear factor kappa B
NNK: nicotine‐derived nitrosamine ketone
NNN: N′‐nitrosonornicotine
PA: polycyclic aromatic hydrocarbon
PFS: progression‐free survival
PVDF: polyvinylidene difluoride
RTK: receptor tyrosine kinase
SCLC: small cell lung cancer
TBST: Tris‐buffered saline Tween‐20
TKI: tyrosine kinase inhibitor

## 1 Introduction

Lung cancer is one of leading cancer types in both males and females, and has a high incidence and mortality worldwide (Siegel *et al*., 2016). Lung cancer is grouped into two major types: non‐small cell lung cancer (NSCLC) and small cell lung cancer (SCLC). NSCLC accounts for 80–85% of lung cancer and can be further classified into three major sub‐types: adenocarcinoma, squamous cell carcinoma and large cell carcinoma. Cigarette smoke and secondhand smoke have been demonstrated as the risk factors for lung cancer (de Groot and Munden, 2012; Kenfield *et al*., 2008; Molina *et al*., 2008). The vast majority (85%) of lung cancer occurs in people aged over 50 years with a history of cigarette smoking (Siegel *et al*., 2016); only 10–15% of cases were non‐smokers (Thun *et al*., 2008).

Cigarette smoke is a mixture including more than 5000 chemicals (Adams *et al*., 1987; Borgerding and Klus, 2005; Talhout *et al*., 2011; Thielen *et al*., 2008). Over 60 carcinogens, including benzene, benzo[α]pyrene (B[α]P), dibenz[a,h]anthracene, catechol, nitromethane, 4‐(methylnitrosamino)‐1‐(3‐pyridyl)‐1‐butanone (NNK), N′‐nitrosonornicotine (NNN), were identified in the mixtures of cigarette smoke (Adams *et al*., 1987; Hecht, 2003; Talhout *et al*., 2011). These carcinogens can be classified into three groups: strong carcinogen, weak carcinogen and co‐carcinogen. Strong carcinogens, including polycyclic aromatic hydrocarbon (PAH) nitrosamines and aromatic amines, can induce tumor formation in immune‐completed laboratory animals after treatment at microgram or milligram level. Weak carcinogens, including acetaldehyde, induce carcinogenesis at a relatively high dose. Co‐carcinogens are chemicals such as nicotine that promote the effects of a carcinogen in the induction of cancer (Hecht, 2012). Among these chemicals, NNK and PAH are two major carcinogens and induce lung cancer formation in fully immune laboratory animals (Hecht, 2003; Huang and Chen, 2011). NNK, a nicotine‐derived nitrosamine ketone, can induce multiple cancer formation, including lung (major), nasal, oral, liver, pancreatic and cervical cancers (Hecht, 2003; Huang and Chen, 2011). It not only mutates or activates oncogenes and tumor suppressor, such as Adrb2, K‐Ras, p53 and TxA_2_ (Huang and Chen, 2011; Huang *et al*., 2011; Kerr *et al*., 2007; Matzinger *et al*., 1995; Zheng and Takano, 2011) but also induces hypermethylation of multiple tumor suppressor gene promoters, such as IGFBP‐3, FHIT, p16^IKK4a^ and RARB (Harada *et al*., 2013; Lin *et al*., 2010). Some studies showed that NNK stimulated Erk signaling pathway and induced cell transformation and proliferation through the EGFR signaling pathway (Askari *et al*., 2005; Laag *et al*., 2006). Benzo[α]pyrene, a PAH, can be metabolized by cytochrome P450s (CYPs), such as CYP1A1, CYP1A2 and CYP1B1 (Chinai *et al*., 2015; Eling *et al*., 1986; Shimada, 2006). B[α]P induces carcinogenesis in mouse models through diolepoxide and radical‐cation mechanism, leading to G to T and G to A mutations in codon 12 of K‐Ras in lung cancer (Mass *et al*., 1993), G to T mutation at codon 13, and A to T mutation at codon 61 in H‐Ras in skin cancer (Chakravarti *et al*., 2008). Tp53 tumor‐suppressor gene is also mutated by B[α]P in skin and lung cancer through G to T transversion mutation in codons 157, 248 and 273 (Denissenko *et al*., 1996; Ruggeri *et al*., 1993).

In addition to genetic alternations associated with tumorigenesis, cigarette smoking behavior was also associated with the insensitivity to EGFR TKIs and poor progression‐free survival (PFS) in different types of cancer patients with EGFR overexpression (Gazdar, 2009; Lee *et al*., 2006; Miller *et al*., 2004; Takano *et al*., 2004). EGFR belongs to the membrane‐bound ErbB (HER) tyrosine kinase receptor family and is important for maintaining cell survival, differentiation and mitogenesis in various cancer types, including NSCLC (Scaltriti and Baselga, 2006). Upon binding with its ligands, EGFR forms homodimers or heterodimers with other ErbB family members including ErbB2, ErbB3 and ErbB4, leading to activation of its tyrosine kinase and downstream signals, such as PI3K/AKT and p42/p44 MAPK pathways (Scaltriti and Baselga, 2006). Overexpression or aberrant activation of EGFR has been demonstrated to cause tumor growth and progression of NSCLC (Dacic *et al*., 2006; Fontanini *et al*., 1995; Normanno *et al*., 2005). Based on this prevailing phenomenon, EGFR is therefore a rational and feasible target for suppression of tumor growth. Gefitinib (ZD1839, Iressa) and erlotinib (OSI‐774, Tarceva) are small molecule EGFR TKIs that function by binding to their ATP‐binding pocket (Noble *et al*., 2004) and have been approved for NSCLC patients. However, they are more effective in certain populations of NSCLC patients: Asian women, never‐smokers and adenocarcinoma patients. The good response of non‐smoker NSCLC patients to EGFR TKIs is associated with activating EGFR mutations (Lynch *et al*., 2004; Paez *et al*., 2004; Pao *et al*., 2004). The majority of these activating mutations are L858R mutation and exon 19 deletion of EGFR (Riely *et al*., 2006; Sharma *et al*., 2007), which showed protein structural alteration and higher binding affinity with EGFR TKIs at their ATP‐binding sites (Carey *et al*., 2006) and rendered this receptor more vulnerable to TKI inhibition. Therefore, these activating mutations have been viewed as a single biomarker to select patients for these TKIs (Lynch *et al*., 2004; Sordella *et al*., 2004; Zhang and Chang, 2008). However, it remains unknown whether and how cigarette smoke influences the sensitivity to EGFR TKIs in lung cancer. Moreover, even though their response rate is lower than with mutant EGFR‐expressing patients, 20–30% of NSCLC patients with amplified wtEGFR can still derive significant survival benefit from an EGFR TKI regimen (Bell *et al*., 2005; Cappuzzo *et al*., 2005; Tsao *et al*., 2005). Furthermore, no EGFR mutation was identified in about 10–20% gefitinib‐responsive patients (Bell *et al*., 2005; Cappuzzo *et al*., 2005; Huang *et al*., 2004; Kim *et al*., 2008; Nishimura *et al*., 2008; Pao *et al*., 2004). These observations suggest that certain NSCLC patients with wtEGFR expression still respond to EGFR TKIs. Therefore, identification of the cigarette smoke‐induced factors causing the insensitivity to these drugs may be helpful not only for the selection of wtEGFR‐expressing responders but also for the development of precise medicine to treat smoker patients.

In this study, we used a non‐biased strategy to explore whether c‐MET upregulation and downstream Akt activation can be elicited by cigarette smoke and its component B[α]P and contribute to EGFR TKI resistance. Our findings not only define the molecular mechanism accounting for the insensitivity to EGFR TKIs in NSCLC patients with a history of cigarette smoking, but also suggest that treatment with c‐MET inhibitors may benefit such patients.

## 2 Materials and methods

### 2.1 Chemicals and reagents

B[α]P was purchased from Sigma‐Aldrich (St. Louis, MO, USA). NNK was purchased from Toronto Research Chemicals (North York, Canada). Crizotinib was purchased from MedChem Express (Monmouth Junction, NJ, USA). Recombinant human hepatic growth (HGF) was purchased from PEPROTECH (Rocky Hill, NJ, USA). All chemicals were dissolved in DMSO and stored at 4 °C or −20 °C.

### 2.2 Cell culture

Non‐small cell lung cancer mucoepidermoid carcinoma NCI‐H292 cell line was cultured in RPMI 1640 (HyClone, Logan, UT, USA) supplemented with 10 mm HEPES, 10 mm sodium pyruvate, 10% FBS (GIBCO‐BRL, Gaithersburg, MD, USA), 100 units·mL^−1^ penicillin, and 100 g·mL^−1^ streptomycin (Thermo Scientific, Waltham, MA, USA). NSCLC adenocarcinoma HCC827 and PC9 lines were cultured in RPMI 1640 (HyClone) supplemented with 10% FBS, 100 units·mL^−1^ penicillin and 100 g·mL^−1^ streptomycin (Thermo). All cells were incubated in a humidified incubator in 5% CO_2_ at 37 °C. HCC827 and PC9 cell lines express EGFR exon19del E746–A750 mutant. These cell lines were gifts from Prof. Mien‐Chie Hung (MD Anderson Cancer Center, Houston, TX, USA).

### 2.3 Preparation of cigarette smoke extract medium

Commercial cigarettes (from Taiwan Tobacco & Liquor Corporation, Taipei, Taiwan) contain nicotine (0.8 mg per cigarette) and tar (10 mg per cigarette). Twenty‐five cigarettes were used to prepare 250 mL of culture medium (RPMI 1640 with or without HEPES serum‐free medium). Smoke from burning 25 cigarettes was inflated into 250 mL culture medium by pump and the pH of medium was adjusted to 7.4. The cigarette smoke extract (CSE) medium was filtrated with a 0.22‐μm filter to remove large particles. The CSE stock medium was stored at −30 °C until use. The stock of CSE medium is 100% (1 cigarette per 10 mL medium) and was diluted to the working concentrations with complete medium (Ishii *et al*., 2001; Ratovitski, 2010; Su *et al*., 1998; Sundar *et al*., 2012; Yang *et al*., 2006).

### 2.4 MTT [3‐(4,5‐dimethylthiazol‐2‐yl)‐2,5‐diphenyl‐tetrazolium bromide] assay

Cells (5 × 10^3^ cells per well) were seeded in 96‐well tissue culture plates and then treated with various EGFR TKIs including erlotinib or gefitinib at the indicated concentrations. Cell viability analysis was measured after treatment for 3 days using MTT (Sigma‐Aldrich), which was dissolved in DMSO. After treatment with TKIs, cells were washed with PBS twice and dissolved in DMSO for the measurement at absorbance 570 nm.

### 2.5 Analysis of phospho‐receptor tyrosine kinase array

Total lysates prepared from both control and B[α]P‐treated cells were subjected to Human Phospho‐Receptor Tyrosine Kinases Array Kit (R&D Systems, Minneapolis, MN, USA), according to the manufacturer's protocol. The changes of phospho‐receptor tyrosine kinase (RTK) expressions were quantified using imagej (NIH, Bethesda, MD, USA).

### 2.6 Gene silencing by lentiviral small hairpin RNA

HEK293T cells (2.4 × 10^6^) were seeded in 6‐cm culture dish in 5 mL DME/F‐12 with 10% FBS and incubated at 37 °C overnight. Three plasmids (pLKO.1‐shRNA or pLenti‐gene:pCMV‐ΔR8.91:pMD.G = 2:2:0.2 μg) were co‐transfected into HEK293T cells with 10.6 μL F2000 transfection reagent. The medium was refreshed with 10% FBS, P/S and 1% BSA after incubation for 6 h. Medium containing lentivirus was collected after 24 h of transfection and stored at −80 °C until use.

Cells (2 × 10^5^ cells per well) were seeded in six‐well tissue culture plate and incubated overnight. Next day, cells were infected with lentivirus at MOI 125 with 8 g·mL^−1^ polybrene (hexadimethrine bromide, Sigma‐Aldrich). After infection for 16–18 h, culture medium was refreshed and 1 μg·mL^−1^puromycin added for selection for 3 days.

### 2.7 Genomic DNA isolation

Genomic DNA was extracted from stable clones with cell lysis buffer (10 mm pH 8.0 Tris‐HCl, 100 mm EDTA and 0.5% SDS), and incubated at 37 °C until completely dissolved, followed by treatment with RNase A at 37 °C for 15–60 min. Ammonium acetate was then added and mixed by vortex, and centrifuged at 14 000 ***g*** for 1 min. Supernatant was transferred to new tubes and 300 μL 100% isopropanol added, shaken 50 times, and centrifuged at 14 000 ***g*** for 1 min. Supernatant was removed and the pellet was washed in 300 μL 70% ethanol, and centrifuged at 14 000 ***g*** for 1 min. The pellet was dried for 15 min and re‐dissolved in TE buffer (pH 8.0). An optical density at 260 (OD_260_) and 280 (OD_280_) were determined for the concentration and purity of samples, respectively.

### 2.8 RNA extraction

Total RNA was extracted from stable clones with TriPure Isolation Reagent (Roche, Mannheim, Germany). First, each sample was mixed with 0.2 mL chloroform per 1 mL TriPure and then centrifuged at 12 000 ***g*** for 15 min to separate the aqueous phase, interphase and organic phases. Total RNA from the aqueous phase was mixed with 0.4–0.6 mL isopropanol at −30 °C for over 30 min. The mixtures were then centrifuged at 12 000 ***g*** for 15 min, washed in 1 mL 75% ethanol twice, and centrifuged at 12 000 ***g*** for 15 min. Finally, supernatant was removed and the RNA pellet dried, followed by re‐dissolution in diethyl pyrocarbonate (DEPC) water at 4 °C overnight.

### 2.9 Reverse‐transcription and polymerase chain reaction

The RT was performed with 1 μg of RNA using MMLV First‐Strand Synthesis Kit (GeneDireX, Las Vegas, NV, USA). The relative mRNA expression of c‐MET was determined using SYBR FAST qPCR kit (KAPA Biosystems, Wilmington, MA, USA). Primer sequences for *c‐MET* used in real‐time quantitative PCR were F’: 5′‐ CCCGAAGTGTAAGCCCAACT‐3′, R’: 5′‐AGGATACTGCACTTGTCGGC‐3′; 18s rRNA: F’: 5′‐CGGCGACGACCCATTCGAAC‐3′, R’: 5′‐GAATCGAACCCTGATTCCCCGTC‐3′; c‐MET genomic exon 2: F’: 5′‐ATAAACCTCTCATAATGAAGGCC‐3′, R’: 5′‐TTTGCTAGTGCCTCTTTACACTC‐3′.

### 2.10 Protein extraction and western blot analysis

Cells were lysed using RIPA lysis buffer with protease and phosphatase inhibitors and centrifuged at 12 000 ***g*** for 30 min. Samples were quantified using Braford assay (Bio‐Rad, Hercules, CA, USA). All samples were separated by 8–12% SDS/PAGE and transferred to 0.45 μm polyvinylidene difluoride (PVDF) membranes (Millipore, Billerica, MA, USA) or 0.22 μm nitrocellulose (NC) membranes (GE Healthcare, Amersham, UK). Non‐specific protein binding was blocked in 5% skim milk with Tris‐buffered saline Tween‐20 (TBST) for 1 h at room temperature. The membranes were hybridized with primary antibodies against phospho‐HER2 (Y1221/1222), phospho‐HER3 (Y1289), phospho‐EGFR (Y1068), phospho‐MET (Y1234/1235), c‐MET, Akt, phospho‐Erk (T202/Y204), Erk (Cell Signaling, Danvers, MA, USA), phospho‐Akt (S473), HER2, HER3 and EGFR (Santa Cruz Biotechnology, Dallas, TX, USA), tubulin, actin (Sigma‐Aldrich) and phosphotyrosine (Merck Millipore, Belmopán, Belize) at 4 °C overnight, followed by incubation with HRP‐labeled secondary antibodies at room temperature for 1 h. The expression of proteins was detected with enhanced chemiluminescence (ECL, GE Healthcare, or Millipore).

### 2.11 Conditioned medium treatment

Cells were cultured and plated on 100‐mm dishes. After 24 h, conditioned media from H292 parental, H292/1%CSE, H292/5%CSE, H292/DMSO and H292/B[α]P 1 μm cells was collected. Fresh conditioned media was centrifuged at 200 ***g*** for 5 min before H292 parental cells were treated with different conditioned media for 6 h. HGF treatment was used as positive control for c‐MET activation. The treated cells were lysed with RIPA lysis buffer to prepare total protein.

### 2.12 ChIP analysis

Cigarette smoke extract‐/B[α]P‐treated H292 cells were fixed with 1% formaldehyde at room temperature for 10 min to cross‐link protein and DNA, and the reaction was stopped by adding glycine. Cross‐linked cells were washed twice with cold PBS and resuspended in 1 mL PBS with protease inhibitor cocktail. These cells were centrifuged at 700 ***g*** for 10 min at 4 °C and the supernatant removed. DNA was digested by treating with micrococcal nuclease (MNase, Thermo Scientific) for an appropriate incubation time at 37 °C after adding nuclear lysis buffer (50 mm Tris‐Cl pH 8.0, 10 mm EDTA, 1% SDS) to break the nuclear membrane. These cell pellets were centrifuged at 900 ***g*** for 5 min at 4 °C and then the supernatant was collected and pre‐cleaned with 60 μL protein A agarose (GE Healthcare, New York, NY, USA) in 900 μL dilution buffer (0.01% SDS, 1% Triton X‐100, 2 mm EDTA, 20 mm Tris‐Cl (pH 8.0), 500 mm NaCl) with protein inhibitor cocktail at 4 °C for 1 h. The supernatants were then centrifuged at 860 ***g*** at 4 °C for 3 min and incubated with primary antibody against MeCP2 (Millipore), 5‐methylcysine (Calbiochem, San Diego, CA, USA) or control IgG at 4 °C overnight. Antibody‐protein‐DNA complex was immunoprecipitated with 60 μL salmon sperm DNA/protein A at room temperature for 2 h. Pellets were eluted by freshly prepared elution buffer (20% SDS and 1 m NaHCO_3_) for 15 min at 65 °C after extensive washing. Nucleic acids were extracted by adding phenol/chloroform/isoamyl alcohol (25 : 24 : 1) (Invitrogen, Carlsbad, CA, USA) and used as the template in SYBR FAST qPCR kit (KAPA Biosystems). Primer sequences for the methylation of *c‐MET* promoter were M1 region (+84 to +484), F′: 5′‐AGCACGTGTCTGTTCGTCCCTG‐3′, R′: 5′‐ CCTTGCCAGCTGTATCACCCTG‐3′; M2 region (−261 to +98), F′: 5′‐ GGACAAACCTAGAGCGACAGGG‐3′, R′: 5′‐ACGCGGCTGGAGTTTGTACC‐3′; M3 region (−563 to −223), F′: 5′‐GCTTTGCGCGGGTGACTTTG‐3′, R′: 5′‐AGCACGTGTCTGTTCGTCCCTG‐3′; M4 region (−710 to –409), F′: 5′‐ATCCGTCCATGCACTCCCAAC‐3′, R′: 5′‐CGGCAAGGTGAAACTTTCTAGG‐3′.

### 2.13 Statistical analyses

Data are presented as the mean ± standard error of the mean (SEM) of three independent results. A two‐tailed *t*‐test was used for most comparisons, with *P *< 0.05 considered significant.

## 3 Results

### 3.1 Cigarette smoke and B[α]P cause the insensitivity of wtEGFR‐expressing NSCLC to EGFR TKIs

**Figure 1:**
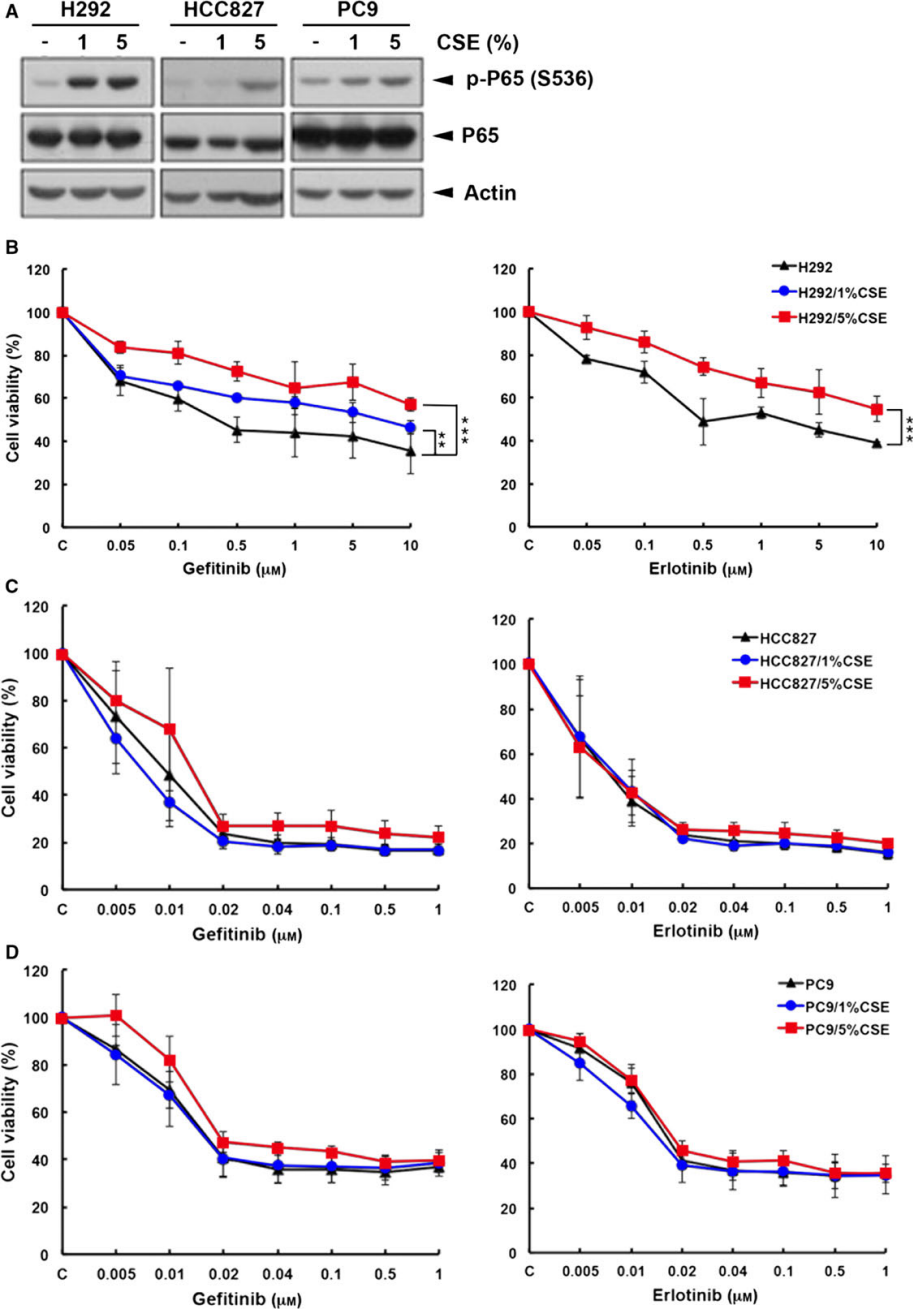
Cigarette smoke extract treatment rendered wtEGFR‐ but not EGFR mutant‐expressing NSCLC more resistant to EGFR tyrosine kinase inhibitors. Whole‐cell extracts prepared from H292/CSE, HCC827/CSE and PC9/CSE. Stable cells were subjected to western blot analysis to determine p65 phosphorylation at ser536 (A). CSE‐treated H292 (B), HCC827 (C) and PC9 (D) were treated with gefitinib (left) or erlotinib (right) for 3 days and their viabilities were determined in MTT assays.

**Figure 2:**
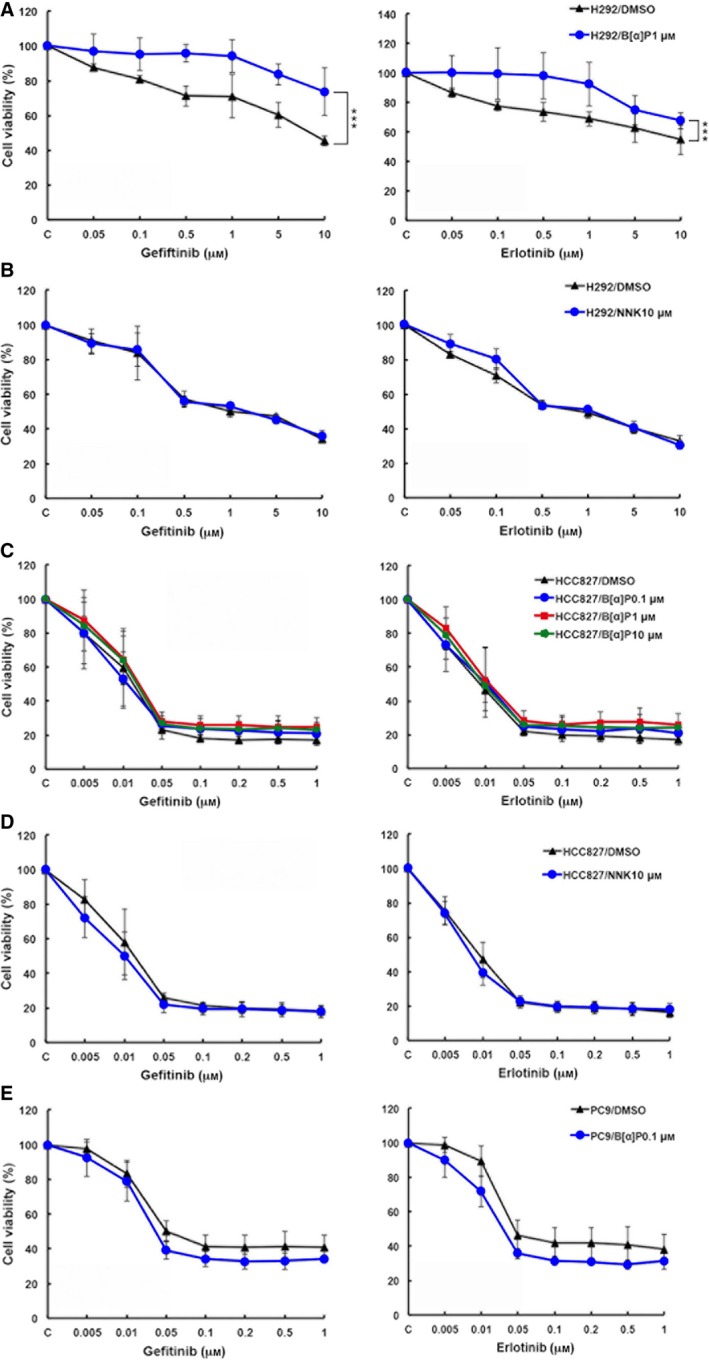
Treatment with B[α]P but not NNK reduces the sensitivity of H292 cells to EGFR tyrosine kinase inhibitors. H292 (A, B), HCC827 (C, D), and PC9 (E) selected with 1 μm B[α]P (A, C, E) or 10 μm NNK (B, D) were treated with gefitinib (left) or erlotinib (right) for 3 days, and their viabilities were determined in MTT assays.

To investigate whether and how cigarette smoke influenced the sensitivity to EGFR TKIs in NSCLC, we first established CSE‐treated clones from wtEGFR‐expressing NCI‐H292 and mutEGFR‐expressing HCC827 and PC9 cells by chronic exposure to 1% CSE or 5% CSE for at least 2 months. Since nuclear factor kappa B (NF‐κB) activation by cigarette smoke has been reported (McMillan *et al*., 2011; Sundar *et al*., 2012; Yang *et al*., 2006), we first examined p65 S536 phosphorylation as a positive control to monitor the proper response of NSCLC cells to CSE treatment (Fig. 1A). The viabilities of these clones under the treatment with EGFR TKIs were then examined in MTT assays. Our data showed that CSE renders H292 cells (Fig. 1B) but not HCC827 or PC9 cells (Fig. 1C,D), more insensitive to both gefitinib and erlotinib as compared with their parental cells. We also tested whether B[α]P or NNK can affect the sensitivity of lung cancer cells to EGFR TKIs. Our data showed that H292/B[α]P stable clones are more resistant to both gefitinib and erlotinib (Fig. 2A), but NNK treatment did not change the sensitivity of H292 cells to EGFR TKIs (Fig. 2B). Similar to the results in HCC827/CSE and PC9/CSE clones, HCC827/B[α]P, HCC827/NNK and PC9/B[α]P stable clones remain sensitive to EGFR TKIs (Fig. 2C–E). These results suggest that B[α]P among the CSE‐derived carcinogens may render wtEGFR‐expressing lung cancer cells more resistant to EGFR tyrosine kinase inhibitors.

**Figure 3:**
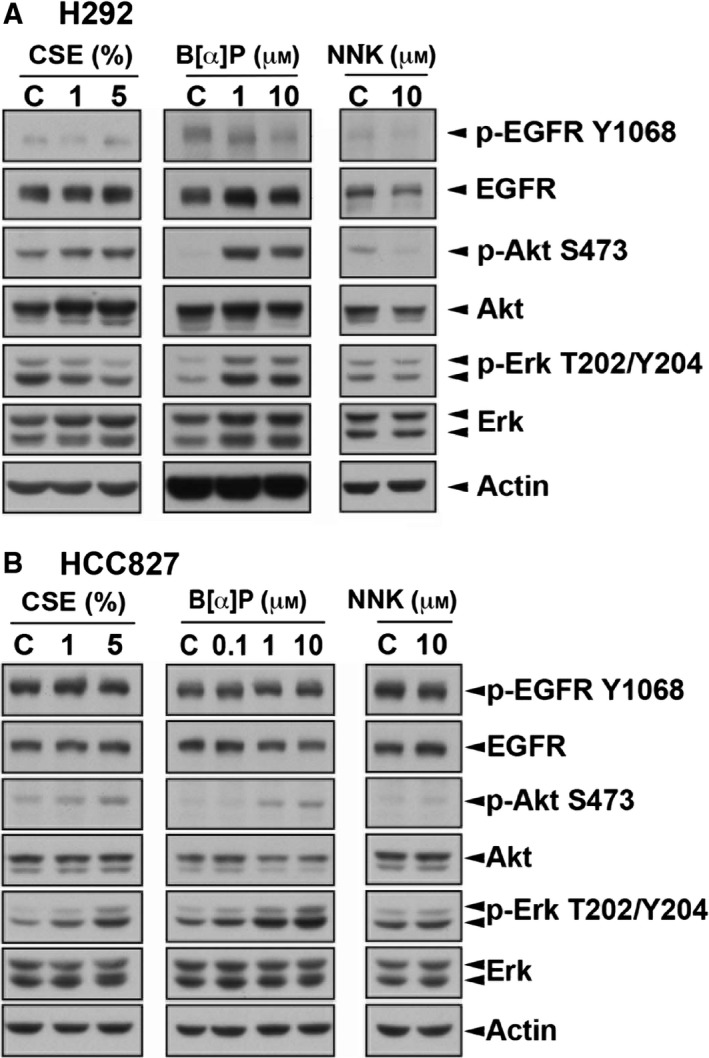
Activities of Akt and Erk were higher in CSE‐ and B[α]P‐selected H292 cells. The activities and protein levels of EGFR and its downstream signaling Akt and ERK were detected in H292 (A) and HCC827 (B) by western blot analysis with indicated antibodies.

We next examined whether EGFR and its downstream signaling pathway in H292, HCC827 and PC9 cells were affected by CSE, B[α]P or NNK. EGFR activity in all CSE‐, B[α]P‐ and NNK‐treated cells was not changed (Fig. 3A,B). However, the Akt activity in wtEGFR‐expressing H292 cells was significantly enhanced in response to CSE and B[α]P but not NNK (Fig. 3A). In EGFR mutant‐expressing HCC827 cells, Akt activation by CSE, B[α]P and NNK was barely detected (Fig. 3B). In both H292 and HCC827 cells, ERK activity was induced by CSE and B[α]P but not NNK (Fig. 3A,B). These findings suggest that the elevation of Akt activity may be critical for CSE‐ and B[α]P‐induced EGFR TKI resistance.

**Figure 4:**
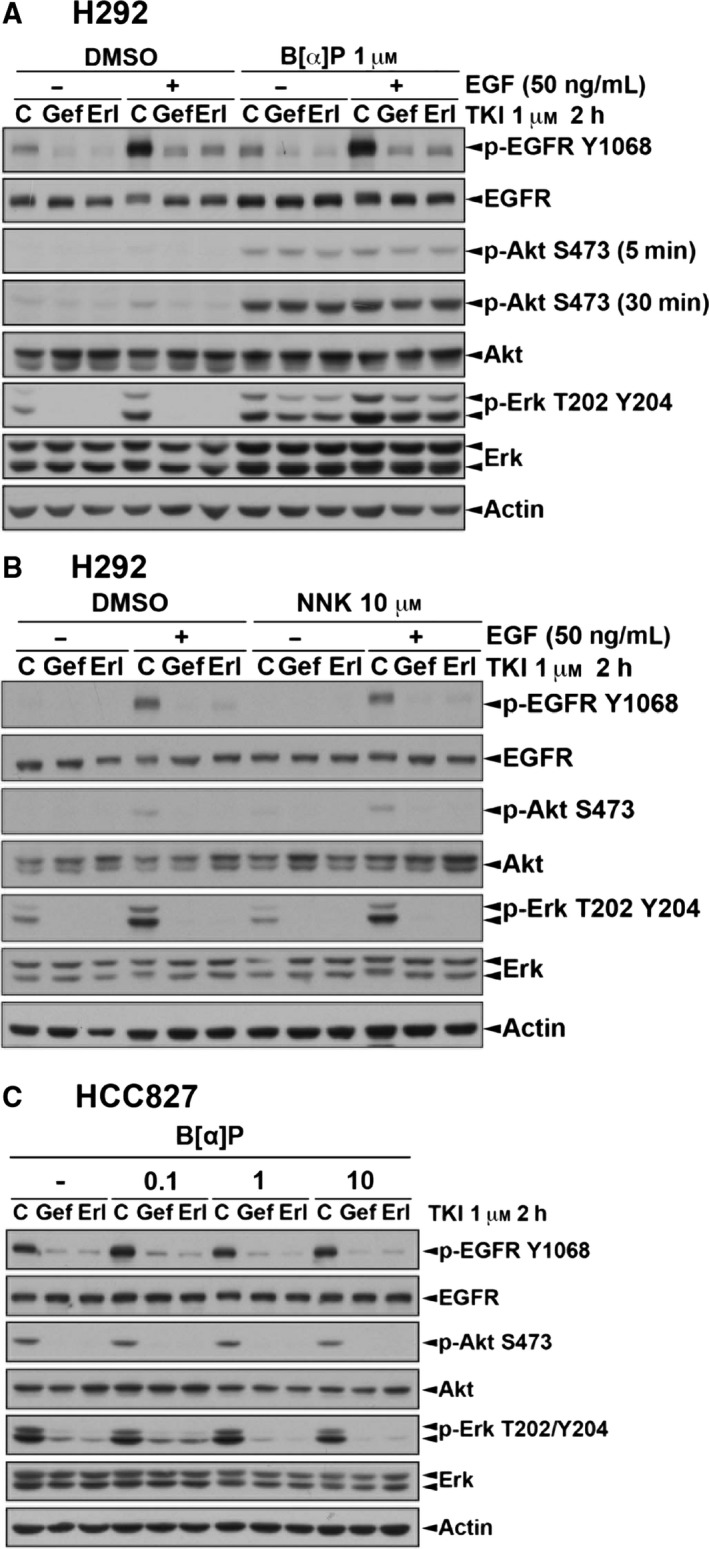
EGFR TKI failed to inhibit activation of Akt and Erk in H292/B[α]P cells but not in HCC827/ B[α]P cells. H292 (A, B) and HCC827 (C) cells selected with B[α]P or NNK were pretreated with 1 μm EGFR TKIs for 2 h followed by 50 ng·mL ^−1^ EGF stimulation as indicated. Whole‐cell extracts were prepared and subjected to western blot analysis to determine the expression of EGFR signaling pathway with indicated antibodies.

We further found that both basal and EGF‐induced EGFR signaling including Akt and ERK activation can be completely inhibited by gefitinib and erlotinib in both parental (Fig. 4A) and NNK‐treated cells (Fig. 4B). In B[α]P‐treated H292 cells, however, the enhanced Akt and ERK kinase activities were not affected by these two EGFR TKIs; in fact, the EGFR activity was dramatically inhibited (Fig. 4A). Both Akt and ERK pathways remain sensitive to EGFR TKIs in CSE‐ and B[α]P‐treated HCC827 stable clones (Fig. 4C), probably due to the stronger oncogene addiction to the activated EGFR mutant in these cells. These data suggested that the increased Akt and ERK activities in response to CSE and B[α]P treatment may result from an alternative signaling and thereby contribute to the insensitivity of wtEGFR‐expressing smoker NSCLC patients to EGFR TKIs.

**Figure 5:**
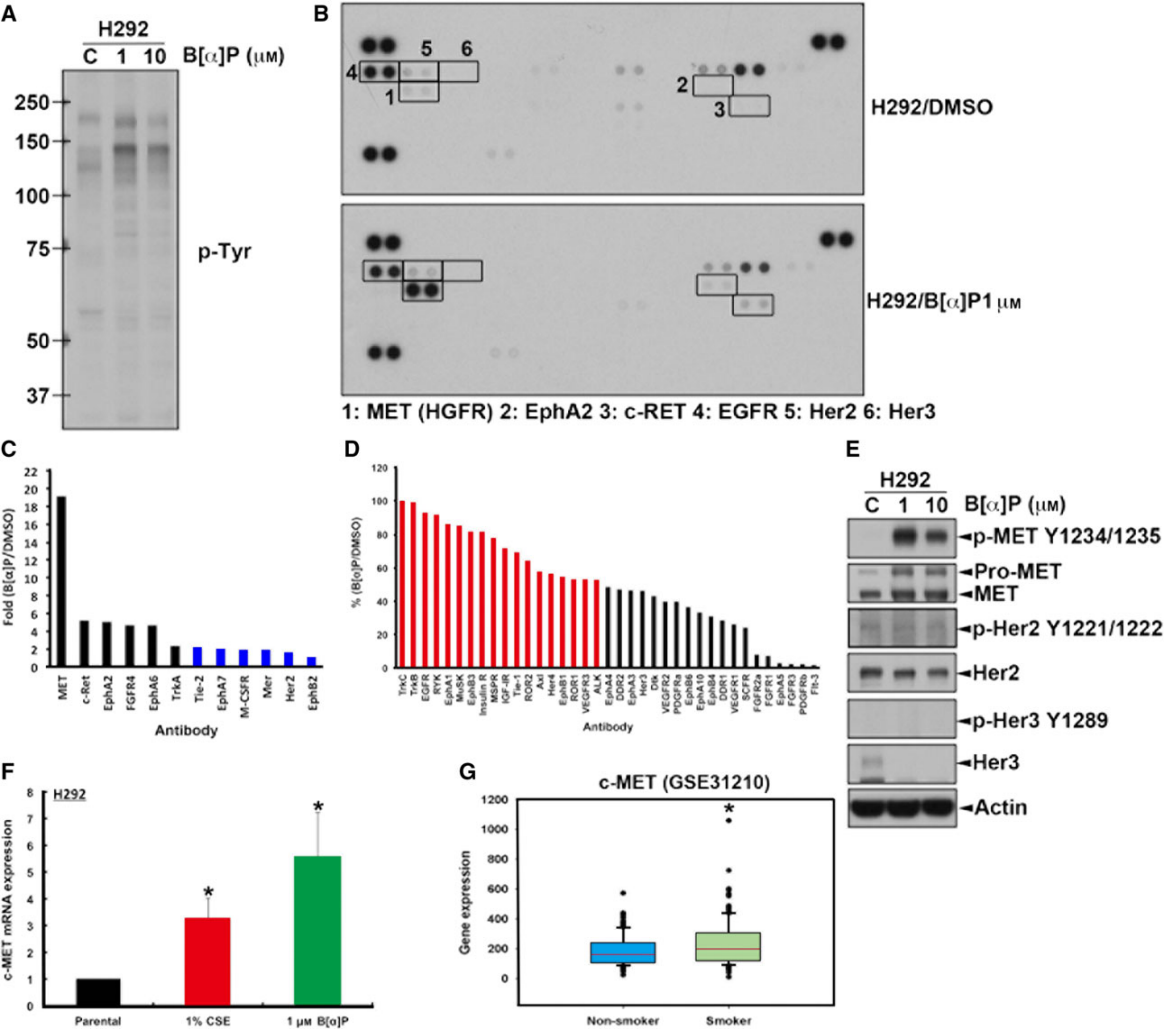
Proto‐oncogene c‐MET expression and activity were higher in H292/B[α]P cells than in control cells. (A) Whole‐cell extract from H292/B[α]P cells was subjected to western blot analysis with anti‐phosphotyrosine antibody. (B) Total lysates prepared from the H292/DMSO and H292/B[α]P were incubated with RTK antibody arrays, and phosphotyrosine was detected by anti‐phospho‐tyrosine‐HRP. The changes in tyrosine phosphorylation of RTK were labeled as indicated. The upregulation (C) and downregulation (D) of RTK tyrosine phosphorylations in H292/B[α]P cells shown in (B) were quantitated using imagej software. (E) The RTK activities and protein levels in H292/B[α]P cells determined by western blot analysis with indicated antibodies. (F) The mRNA level of c‐MET in indicated cells was detected by q‐RT‐PCR. (G) The c‐MET mRNA levels in non‐smoker and smoker NSCLC patients were analyzed from the GSE31210 GEO dataset.

To examine whether this resistance was caused by EGFR T790M mutation, which has been reported as a secondary mutation after treatment with EGFR TKIs (Mitsudomi and Yatabe, 2007; Sharma *et al*., 2007), cDNA of EGFR was extracted and prepared from these CSE‐ and B[α]P‐treated clones and sequenced. Although G to A mutation at codon 787 was found, this substitution is a silent mutation and did not alter the amino acid sequence. However, no mutation at codon 790 was found, suggesting that the resistance of CSE‐ and B[α]P‐treated H292 cells to EGFR TKI was not caused by EGFR T790M mutation (Fig. S1). We further investigated whether other receptor tyrosine kinases were involved in the elevation of Akt and ERK activities by B[α]P. We examined the expression profile of protein tyrosine phosphorylation in B[α]P‐treated cells in western blot analysis with anti‐phosphotyrosine antibody. Interestingly, an increased tyrosine phosphorylation signal around 150 kDa was detected in the B[α]P‐treated H292 cells (Fig. 5A). Therefore, we suggest that certain RTKs might be activated in response to B[α]P treatment to mediate the activations of AKT and ERK signaling. To further identify which RTK is activated by B[α]P, total lysates prepared from both parental and B[α]P‐treated cells were subjected to RTK antibody array analysis. The results showed that MET tyrosine phosphorylation is dramatically increased in the H292/B[α]P cells. However, the phosphorylation status of EGFR, HER2 and HER3 was not changed by B[α]P (Fig. 5B). As shown in Fig. 5C, the activation of c‐MET was 19‐fold higher in H292/B[α]P cells than in parental cells in the quantitated data from RTK antibody array analysis. Other RTKs, including c‐RET and EphA2, were also activated in response to B[α]P. However, IGF‐IR, Axl and Alk, which were known to play some roles in the acquired resistance to EGFR TKI (Bae *et al*., 2015; Guix *et al*., 2008; Maione *et al*., 2015), were not activated by B[α]P and may not be involved in the insensitivity of smoker NSCLC to EGFR TKI (Fig. 5D).

### 3.2 CSE and B[α]P enhance the oncogene addiction of wtEGFR‐expressing NSCLC to c‐MET

**Figure 6:**
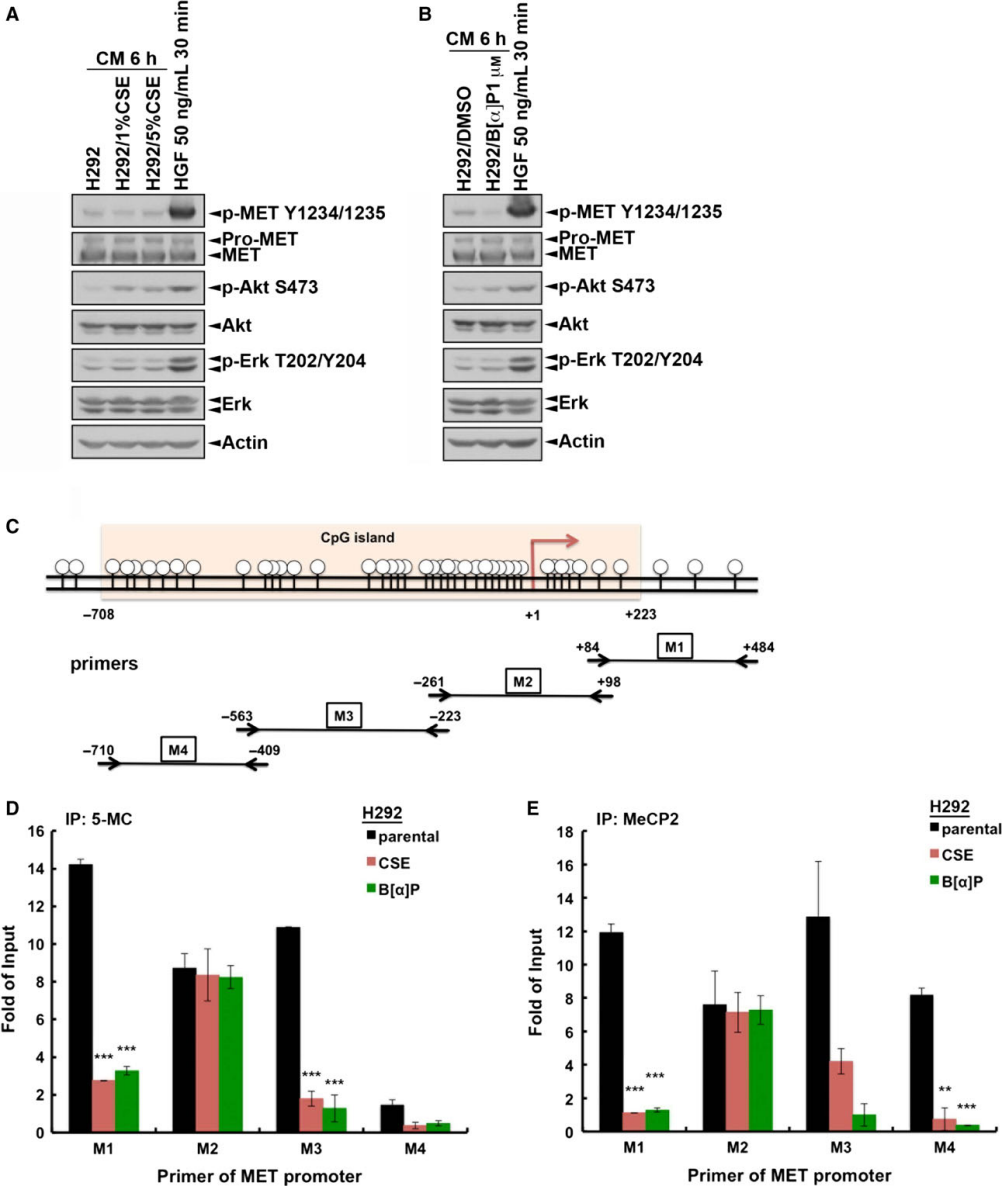
Cigarette smoke extract and B[α]P induced c‐MET expression through promoter de‐methylation. Parental H292 cells were treated with conditioned medium of (A) H292/CSE or (B) H292/B[α]P cells for 6 h. The whole‐cell lysates were then prepared and subjected to western blot analysis with indicated antibodies. (C) The CpG island on *c‐MET* promoter and primer sets used in ChIP analysis were illustrated. Parental H292 cells and their CSE and B[α]P clones were subjected to ChIP analysis with 5‐methylcytosine (5‐mC) (D) or anti‐MeCP2 (E) antibodies followed by quantitative PCR with primers targeting c‐MET promoter. ‘Fold of Input’ is expressed as the ratio between the amount of total immunoprecipitated DNA (bound) and the amount of input DNA. Data are expressed as means ± SD. **P < 0.005; ***P < 0.001.

To prove that cigarette smoke renders NSCLC cancer cells more resistant to EGFR TKI through induction of c‐MET signaling, we first confirmed the c‐MET activation in H292/B[α]P cells by western blot analysis. As shown in Fig. 6E, c‐MET tyrosine phosphorylation was indeed dramatically increased in B[α]P‐treated cells, and total c‐MET expression is also induced. By contrast, NNK suppressed c‐MET expression (Fig. S2). In addition, exposure to CSE, B[α]P or NNK had no dramatic effect on c‐MET phosphorylation or protein expression in PC9 and HCC827 cells (Fig. S2). The tyrosine phosphorylation of HER2 and HER3 was also determined, but no difference between B[α]P‐treated cells and control cells was detected (Fig. 5E). We further checked the mRNA level of c‐MET in CSE‐ and B[α]P‐selected cells by RT‐qPCR analysis and found an elevation of c‐MET mRNA expression in response to these treatments (Fig. 5F), suggesting that B[α]P transcriptionally upregulates c‐MET gene expression. In support of this finding, the published microarray dataset of 226 lung cancer patients (GSE31210) also showed that cMET mRNA is higher in the lung tumor tissue in smoker than in non‐smoker patients (Fig. 5G).

In addition to c‐MET gene expression, the induction of c‐MET activity may also be due to the stimulation by autocrine ligand, which could be induced by CSE and B[α]P and released into the medium. According to Bogler's model, autocrined HGF activates c‐MET signaling to enhance HGF expression in ΔEGFR‐expressing GBM cells (Garnett *et al*., 2013). We therefore collected the conditioned medium (CM) from control cells or from CSE or B[α]P stable cells to treat parental H292 cells, followed by examination of c‐MET signaling. Unlike the stimulation by HGF, however, the conditioned medium from CSE‐ (Fig. 6A) or B[α]P‐treated (Fig. 6B) cells did not induce c‐MET activity in parental H292 cells. Therefore, we excluded the possibility that the CSE‐ or B[α]P‐induced c‐MET activity is due to the autocrine ligand stimulation. There is a CpG island (from –708 to +233) on the promoter of c‐MET gene, and methylation of this region suppressed c‐MET transcription through recruitment of MeCP2 (Morozov *et al*., 2008; Plummer *et al*., 2013). To examine whether CSE and B[α]P upregulated c‐MET expression by modulating the DNA methylation status on c‐MET promoter, ChIP was performed with anti‐5‐methylcytosine (5‐mC) and anti‐MeCP2 antibodies, followed by quantitative PCR with four different primer sets for c‐MET promoter (as illustrated in Fig. 6C). The results showed that all four regions of c‐MET promoter were detectable in both anti‐5mC and anti‐MeCP2 immunoprecipitates of parental H292 cells (Fig. 6D,E). More importantly, treatments with CSE and B[α]P can suppress cytosine methylation and MeCP2 recruitment on c‐MET promoter in M1, M2 and M4 regions. These results suggest that CSE and B[α]P may upregulate c‐MET expression through removal of DNA methylation on its promoter.

**Figure 7:**
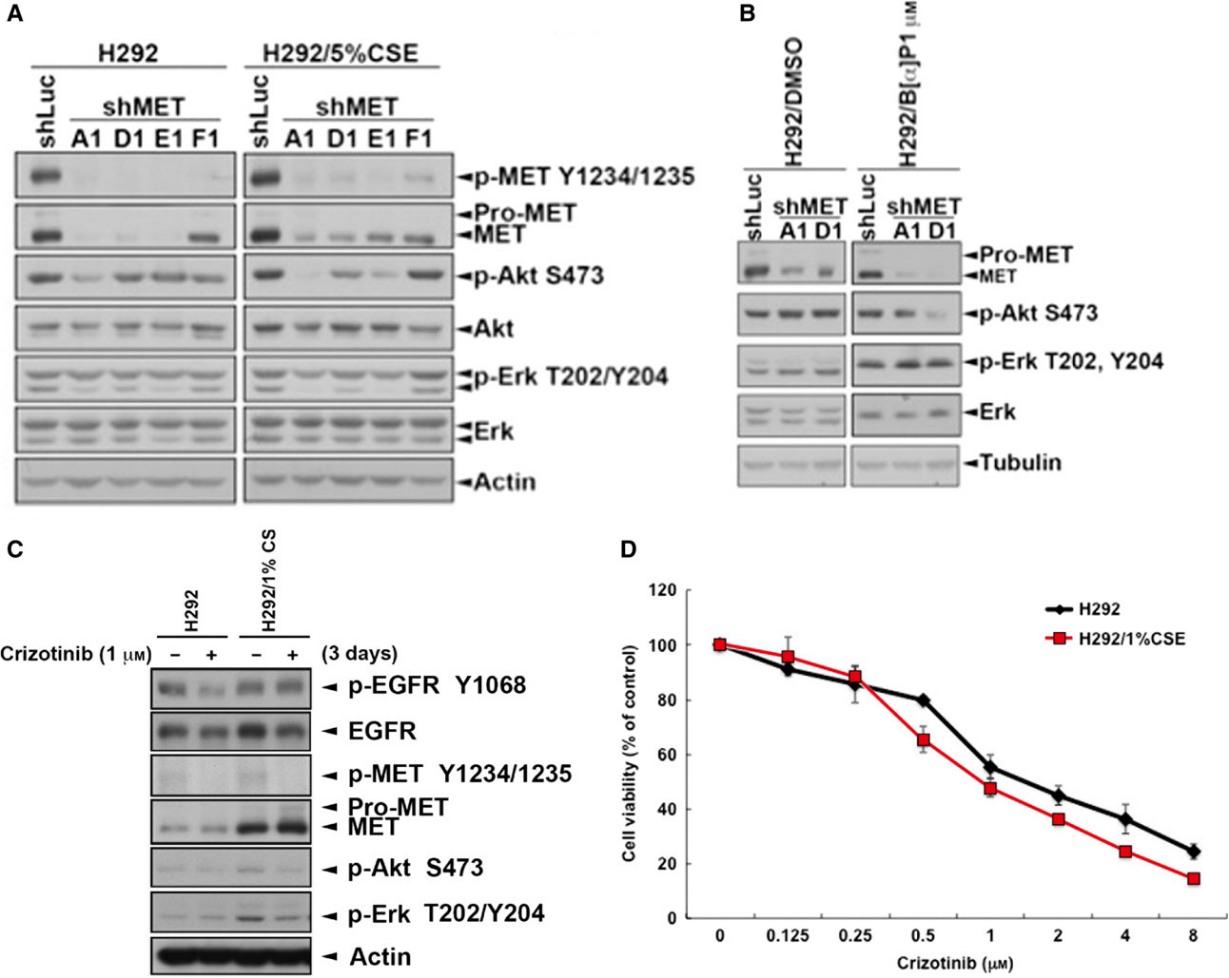
The activity of Akt was reduced by inhibition of c‐MET in H292/CSE and H292/B[α]P cells. c‐MET expression was knocked down by shRNA in H292/CSE (A) and H292/B[α]P (B) cells for 3 days. (C,D) These stable clones were treated with 1 μm crizotinib for 3 days. Total lysates were collected and subjected to western blots with indicated antibodies (C) and the cell viability was analyzed in MTT assay (D).

To further investigate whether the increased c‐MET expression and activity is responsible for Akt and Erk activation and cell viability of CSE‐ and B[α]P‐treated cells, expression and kinase activity of c‐MET were down‐regulated by shRNA and pharmacological inhibitor, respectively. As shown in Fig. 7, Akt but not ERK activity was reduced in CSE‐ and B[α]P‐treated cells but not in parental cells after silencing c‐MET expression (Fig. 7A,B) and treatment with c‐MET inhibitor crizotinib (Fig. 7C). In addition, inhibition of c‐MET by crizotinib also showed a suppressive effect on the viability of both CSE‐treated and parental cells (Fig. 7D). These results indicated that cigarette smoke increased the PI3K/Akt survival pathway for insensitization to EGFR TKIs through enhancing c‐MET expression and activity.

**Figure 8:**
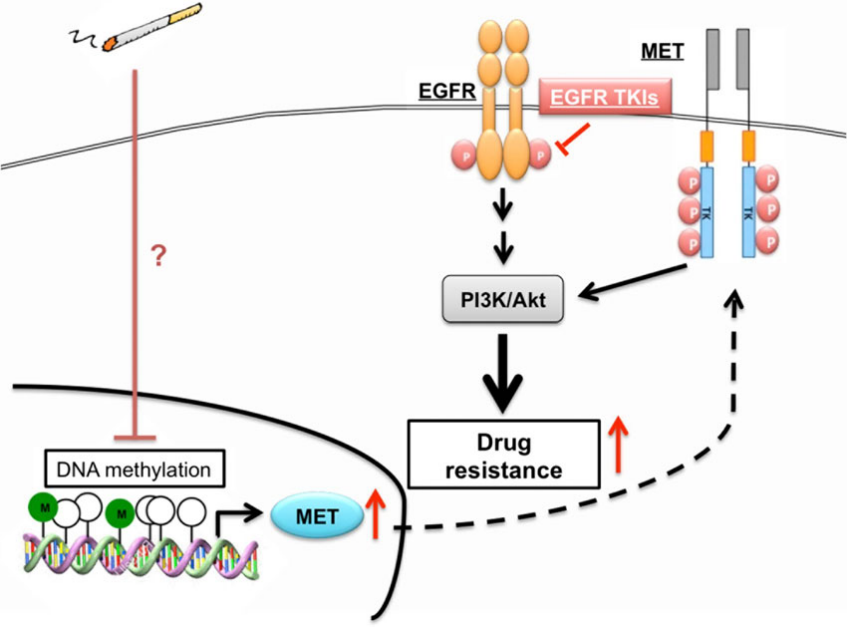
Current hypothetic model of this study. Cigarette smoke de‐represses c‐MET expression through reduction of promoter methylation. The induced c‐MET induced drug resistance to EGFR TKIs by maintaining Akt activity in wtEGFR‐expressing lung cancer cells.

In conclusion, we demonstrated that cigarette smoke extract and its derived carcinogen, benzo[α]pyrene, activated proto‐oncogene MET signaling through ligand‐independent pathway to dominate Akt activation, which may thereby lead to the EGFR TKI resistance in wild‐type EGFR‐expressing NSCLC cell line but not in mutant EGFR‐expressing NSCLC cell lines (Fig. 8). Therefore, our findings not only indicate the possibility of c‐MET activation as the molecular mechanism underlying cigarette smoke‐related EGFR TKI resistance, but also suggest that c‐MET inhibitors may benefit NSCLC patients who smoke, who harbor wtEGFR‐expression.

## 4 Discussion

Cigarette smoke is an important risk factor of lung cancer progression. Clinical studies showed that lung cancer patients who are former or current smokers, are more resistant to EGFR TKIs (Organ and Tsao, 2011). Induction of cytochromes CYP1A1/1A2 by cigarette smoking has been hypothesized to alter erlotinib pharmacokinetics (Hamilton *et al*., 2006). However, our data showed that EGFR TKIs almost abolished the EGFR activity in CSE/B[α]P‐treated cell lines, suggesting that drug metabolism may not contribute to the EGFR TKI insensitization of NSCLC by cigarette smoke. In this study, we found that CSE and its carcinogen B[α]P may render wtEGFR‐expressing lung cancer cells more insensitive to EGFR TKI through activation of the c‐MET/Akt signaling axis. However, CSE/B[α]P‐induced c‐MET activation was not observed in HCC827 or PC9 cells and did not cause EGFR TKI resistance. Consistent to our findings, c‐MET amplification was frequently detected in wtEGFR, but not in mutant EGFR‐expressing NSCLC patients, and is associated with a poor prognosis (Song *et al*., 2017). Since HCC827 cells express EGFR activating mutant, which dominantly controls pro‐survival Akt signal, cigarette smoke may only switch the oncogene addition from the EGFR to MET pathway in wtEGFR‐expressing NSCLC cancer cells. Interestingly, the activation of β1‐adrenergic receptor (β1‐AR), a G protein‐coupled receptor, has been reported to mediate the NNK‐induced IGF‐1R activation and tumorigenesis of human bronchial epithelial cells (Min *et al*., 2016). β‐AR2 was recently demonstrated to cause EGFR TKI resistance through inactivation of LKB1 and induction of interleukin‐6 expression in NSCLC harboring EGFR activating mutation (Nilsson *et al*., 2017). However, our data did not show a change in TKI sensitivity of H292 and HCC827 cells caused by NNK (Fig. 2). These studies suggest that NNK‐activated β1‐AR may be involved in NSCLC carcinogenesis but not its EGFR TKI resistance.

The receptor‐tyrosine kinase c‐MET is the receptor for hepatocyte growth factor (HGF) and activates a wide range of different cellular signaling pathways, including PI3K/Akt, MAPK, JNK, PKC and FAK, which contribute to proliferation, motility, migration and invasion (Organ and Tsao, 2011). In the network enrichment analysis, c‐MET has been identified as a hub which plays key roles in cancer initiation and progression of cigarette smoking‐associated NSCLC (Pazhouhandeh *et al*., 2017). Activation of the HGF/c‐MET signaling pathway has also been found to be associated with a poor prognosis in various solid tumors including NSCLC (Guo *et al*., 2014; Zhang *et al*., 2015), indicating a predictive value for this disease. The critical role of crosstalk between the c‐MET and ErbB family in the development of resistance to cancer therapeutics has been well elucidated (Lai *et al*., 2009). The increased expression and activation of c‐MET in response to EGFR TKIs (Engelman *et al*., 2007) and cytotoxic anticancer agent (Ozasa *et al*., 2014) contribute to drug resistance in lung cancer cells through compensatory enhancing of Akt signaling. Suppression of c‐MET expression by siRNA or inhibition of c‐MET activation by pharmacological inhibitor re‐sensitized the resistant cells to these anticancer agents (Ozasa *et al*., 2014), indicating the importance of c‐MET overexpression in the development of acquired resistance. In support of our finding, NSCLC patients with soluble c‐Met levels > 766 ng·mL^−1^ have shown significant short median PFS after EGFR‐TKI treatment (Gao *et al*., 2016).

Mechanisms resulting in constitutive or prolonged activation of c‐MET during tumor growth or cancer progression include the occurrence of specific genetic lesions, including translocations, gene amplifications and activating mutations; and transcriptional upregulation of the c‐MET protein in the absence of gene amplification or via ligand‐dependent autocrine or paracrine mechanisms (Danilkovitch‐Miagkova and Zbar, 2002). High c‐MET gene copy number is associated with poor survival in NSCLC, ovarian clear cell adenocarcinoma and gastric cancer (Beau‐Faller *et al*., 2008; Cappuzzo *et al*., 2009; Chen *et al*., 2011; Go *et al*., 2010; Park *et al*., 2012; Shi *et al*., 2012; Yamashita *et al*., 2013). In oral lichen planus, the expression of c‐MET was found to be higher in smokers than non‐smokers (Kłosek *et al*., 2011). However, few studies reported how cigarette smoke increases c‐MET expression and activity. Previous studies suggested that mutation of c‐MET at the sema or juxtamembrane domain, including N375S, R988C, T1010I, S1058P or splicing mutation, caused c‐MET activation (Lawrence and Salgia, 2010; Onozato *et al*., 2009). In Soundararajan's study, most of the N375S mutation carriers were eastern Asian, male, squamous and smokers (Johnson *et al*., 2012; Krishnaswamy *et al*., 2009). Based on these studies, the exon2 (sema domain) and exon 14 (juxtamembrane domain) of c‐MET were amplified and sequenced. These mutations did not occur after treatment with CSE and B[α]P (data not shown). Gene amplification was also not observed in the PCR analysis (data not shown). Therefore, we ruled out the possibility that CSE‐ or B[α]P‐induced c‐MET activation is due to the mutation or amplification of *c‐MET* gene. Activation of MET classically is through binding with its ligand, HGF, inducing receptor homodimerization, and then the C‐terminal tail transphosphorylation, leading to the initiation of downstream signals (Feng *et al*., 2012). Chen *et al*. (2006) suggested that cigarette smoke could overexpress HGF in type II pneumocytes and lung cancer cells. HGF‐dependent MET activation via endocrine, paracrine or autocrine signaling had been observed in lung cancer (Feng *et al*., 2012). In our study, however, the condition medium from benzo[α]pyrene‐treated clone did not stimulate MET tyrosine phosphorylation, suggesting that activation of MET in these cells is not through the HGF autocrine‐dependent pathway.

Several microRNAs, including miR‐613 (Li *et al*., 2016b), miR‐138 (Li *et al*., 2016a), miR‐206 (Zheng *et al*., 2015), miR‐185 (Fu *et al*., 2014) and miR‐101 (Hu *et al*., 2013), have been demonstrated to target c‐MET for cancer proliferation and progression. Cigarette smoke globally downregulates microRNA expression in human alveolar macrophages (Graff *et al*., 2012). Further studies are required to demonstrate the possibility that CSE induces c‐MET expression through downregulation of microRNAs. In addition, physical protein interactions with other receptor tyrosine kinases have been reported to contribute to MET activation (Cassinelli *et al*., 2009; Ju and Zhou, 2013; Tanizaki *et al*., 2011). An association between integrin beta1 and c‐MET has also been reported to mediate EGFR TKI resistance through activation of the c‐MET signaling pathway in NSCLC (Ju and Zhou, 2013). Further investigations are required to examine the possibility that these potential mechanisms underlie benzo[α]pyrene‐induced MET activation.

In a meta‐analysis, targeting c‐MET therapies has been found to improve progression‐free survival (PFS) and disease control rate (DCR) in advanced or metastatic NSCLC patients. However, c‐MET inhibitors did not show the therapeutic benefits on their overall survival and objective response rate (Ye *et al*., 2016). It would be interesting to analyze the impact of cigarette smoke on the therapeutic efficacy of c‐MET inhibitor in NSCLC patients. In our results, when MET expression was silenced in CSE‐ and B[α]P‐treated cells, the activity of Erk is not affected by MET inhibition. This observation suggests that elevation of other unidentified driver genes may be responsible for the increased Erk signals, which may thereby become an obstacle to the treatment of NSCLC patients who are smokers, even if MET inhibitors are used. Therefore, further investigation of the underlying mechanisms of B[α]P‐induced ERK activation are indicated.

## 5 Conclusions

In this study, our data showed that cigarette smoke and its derivative B[α]P reduce the sensitivity of wtEGFR‐ but not EGFR mutant‐expressing NSCLC cells to EGFR TKIs. c‐MET is upregulated and activated by CSE and B[α]P for the compensatory Akt activation and resistance to EGFR inhibitors. These findings not only explain the critical role of c‐MET in the primary resistance of NSCLC patients who are smokers, to EGFR TKIs, but also suggest that targeting c‐MET may benefit such patients.

## Author contributions

Study concepts: WCH and CYT. Study design: WCH and CMC. Data acquisition: FJC, SLW, YCH, BWW, YHH and YJC. Quality control of data and algorithms: CHC and TCH. Data analysis and interpretation: ISH and CHY. Statistical analysis: FJC, SLW and YLY. Manuscript preparation: WCH, FJC, SLW and CYT. Manuscript editing: CYT. Manuscript review: WCH. All authors read and approved the final manuscript.

## Supporting information


**Fig. S1.** EGFR mutations were not found in CSE‐ or B[α]P‐treated cells. The EGFR cDNA in H292/1%CSE (A), H292/5%CSE (B) or H292/B[α]P cells (C) were prepared by RT‐qPCR, and sequenced.

**Fig. S2.** c‐MET phosphorylation and protein expression in NSCLC cells in response to cigarette smoke and its oncogene ingredients. Total protein lysate of H292, PC9 and HCC827 cells and their CSE, B[α]P and NNK‐treated clones were prepared and subjected to western blot analysis with indicated antibodies.
