# Supplementary figures and images for: Cigarette smoke enhances oncogene addiction to c‐MET and desensitizes EGFR‐expressing non‐small cell lung cancer to EGFR TKIs
Source: Mol Oncol. 2018 Apr 14;12(5):705–23. doi: 10.1002/1878-0261.12193 (PMC5928373; doi:10.1002/1878-0261.12193)

Figure S1

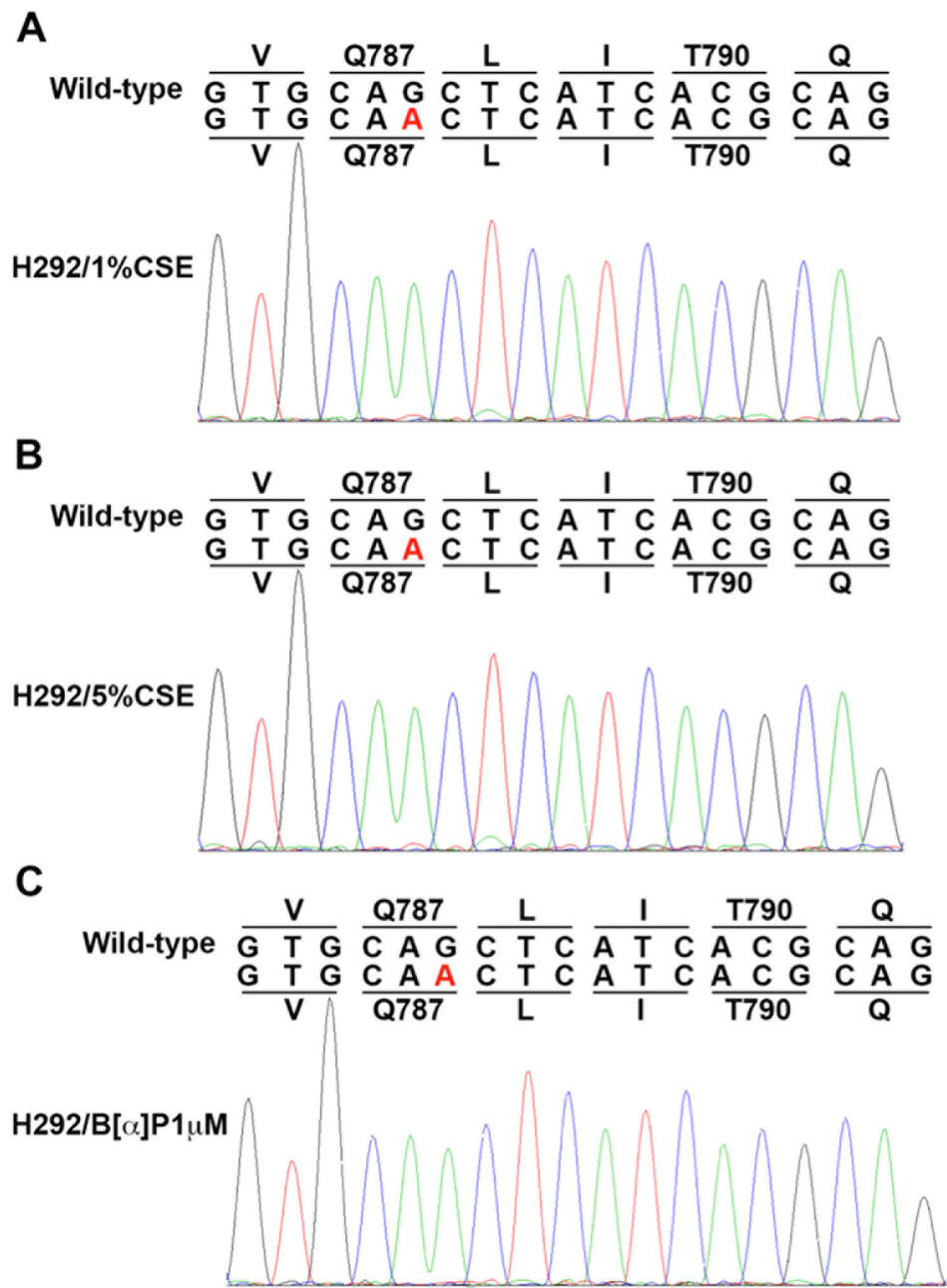

Supplement: Supplementary file 1 — Fig. S1. EGFR mutations were not found in CSE‐ or B[α]P‐treated cells. The EGFR cDNA in H292/1%CSE (A), H292/5%CSE (B) or H292/B[α]P cells (C) were prepared by RT‐qPCR, and sequenced. [file MOL2-12-705-s001.pdf]

Figure S2

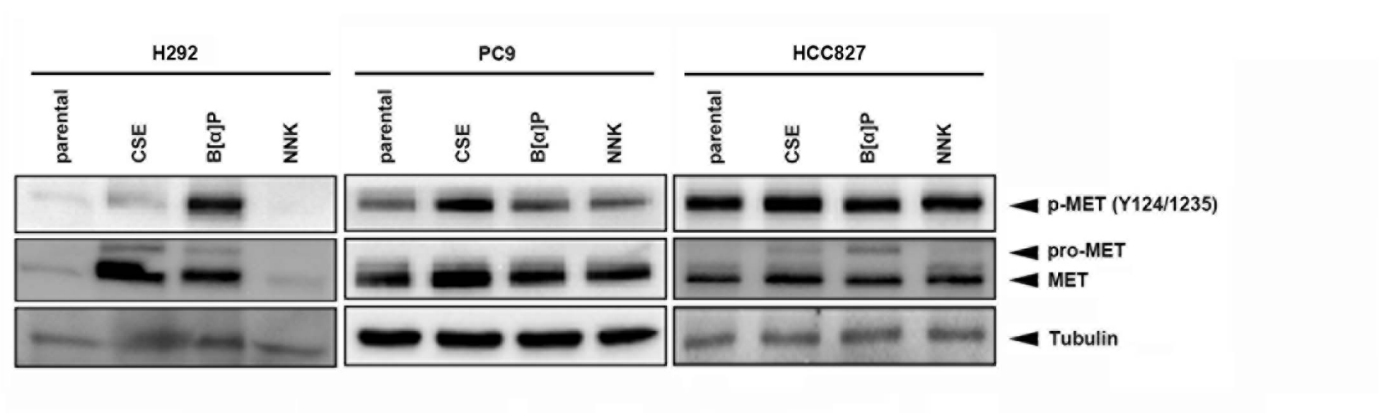

Supplement: Supplementary file 2 — Fig. S2. c‐MET phosphorylation and protein expression in NSCLC cells in response to cigarette smoke and its oncogene ingredients. Total protein lysate of H292, PC9 and HCC827 cells and their CSE, B[α]P and NNK‐treated clones were prepared and subjected to western blot analysis with indicated antibodies. [file MOL2-12-705-s002.pdf]
